# Structural Impact of the E113Q Counterion Mutation on the Activation and Deactivation Pathways of the G Protein-coupled Receptor Rhodopsin

**DOI:** 10.1016/j.jmb.2008.04.055

**Published:** 2008-06-27

**Authors:** Jörg Standfuss, Ekaterina Zaitseva, Mohana Mahalingam, Reiner Vogel

**Affiliations:** 1 Structural Studies Division, MRC Laboratory of Molecular Biology, Hills Road, Cambridge CB2 0QH, UK; 2 Arbeitsgruppe Biophysik, Institut für Molekulare Medizin und Zellforschung, Albert-Ludwigs-Universität Freiburg, Hermann-Herder-Str. 9, 79104 Freiburg, Germany

**Keywords:** GPCR, G protein-coupled receptor, FTIR, Fourier-transform infrared, PSB, protonated Schiff base, SB, deprotonated Schiff base, H, transmembrane helix, EC, extracellular loop, PC, phosphatidyl choline, DDM, dodecyl maltoside, HOOP, hydrogen-out-of-plane, H/D, hydrogen/deuterium, infrared spectroscopy, membrane protein, visual pigment, G protein-coupled receptor, signal transduction

## Abstract

Disruption of an interhelical salt bridge between the retinal protonated Schiff base linked to H7 and Glu113 on H3 is one of the decisive steps during activation of rhodopsin. Using previously established stabilization strategies, we engineered a stabilized E113Q counterion mutant that converted rhodopsin to a UV-absorbing photoreceptor with deprotonated Schiff base and allowed reconstitution into native-like lipid membranes. Fourier-transform infrared difference spectroscopy reveals a deprotonated Schiff base in the photoproducts of the mutant up to the active state Meta II, the absence of the classical pH-dependent Meta I/Meta II conformational equilibrium in favor of Meta II, and an anticipation of active state features under conditions that stabilize inactive photoproduct states in wildtype rhodopsin. Glu181 on extracellular loop 2, is found to be unable to maintain a counterion function to the Schiff base on the activation pathway of rhodopsin in the absence of the primary counterion, Glu113. The Schiff base becomes protonated in the transition to Meta III. This protonation is, however, not associated with a deactivation of the receptor, in contrast to wildtype rhodopsin. Glu181 is suggested to be the counterion in the Meta III state of the mutant and appears to be capable of stabilizing a protonated Schiff base in Meta III, but not of constraining the receptor in an inactive conformation.

## Introduction

The visual pigment rhodopsin is by far the best characterized member of the family of G protein-coupled receptors (GPCRs).^1^ In contrast to other GPCRs, rhodopsin is not activated by diffusible ligands, but by photoisomerization of its retinal chromophore, which is covalently bound to Lys296 on H7 in the transmembrane domain of the protein by a protonated Schiff base. This isomerization from 11-*cis* to all-*trans* switches the light-sensing ligand from an inverse agonist to a full agonist and triggers the activation of the receptor. Rhodopsin offers the unique possibility of using light as a trigger to explore the single steps of the activation of a GPCR on a molecular level by a variety of biophysical methods. It allows interpretation of the data on the basis of solid crystallographic structures, which are available for the dark state,^2–5^ and for some of the inactive photo intermediates,^6,7^ despite the fact that a reliable crystal structure of the active state, Meta II, is still lacking.

**Fig. 1:**
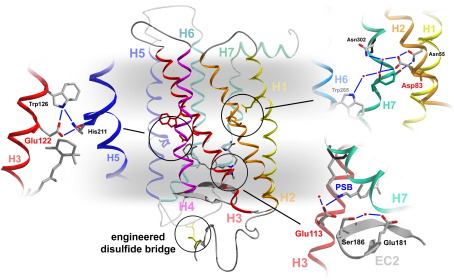
Molecular structure of the dark state of rhodopsin. The model shows the helix bundle of stabilized rhodopsin with the engineered disulfide bridge between residues 2 and 282,^3^ stabilizing the interaction between the N terminus and the extracellular loop 3. Close-ups show the hydrogen-bonded network extending from the Glu113/PSB salt-bridge to Glu181 on EC2, the Glu122 network between H3 and H5 next to the ring of retinal, and the water-bridged Asp83 network between H1, H2, and H7, which extends further to Trp265 on H6 via an additional water molecule (the cytoplasmic side is upward).

The inactive conformation of the dark state is stabilized mainly by two constraints; namely, by the geometry of the 11-*cis* ligand and by a salt bridge in the transmembrane part of the receptor. This salt bridge is formed between the protonated retinal Schiff base (PSB) and a protein counterion consisting of Glu113 on transmembrane helix H3,^8–10^ which appears to be further stabilized by the negative charge of Glu181 on the extracellular loop 2 (EC2) (Fig. 1).^11^ After photoisomerization of the chromophore, the receptor evolves through a series of inactive photointermediates, Batho, BSI, Lumi, and Meta I, which are accessible in time-resolved or cryo-trapping experiments, to the active state Meta II. During this evolution, the salt bridge to the PSB is weakened and the function of the counterion shifts gradually from Glu113 to Glu181 in the transitions to Lumi and Meta I.^11,12^ It is broken in the conformational transition to Meta II by a proton transfer from the PSB to Glu113,^13^ leading to a deprotonated retinal Schiff base (SB) in Meta II and a shift of the absorption maximum from the visible range to 380 nm. The transition from inactive Meta I to Meta II further involves uptake of a solute proton by a cytoplasmic group,^14^ which renders the conformational equilibrium between Meta I and Meta II pH-dependent.^15^ These two protonation switches are coupled during rhodopsin activation and this coupling was shown to be mediated by certain groups of the all-*trans* retinal ligand, such as the 9-methyl group on the polyene and the ionone ring.^16^

The PSB-Glu113 salt bridge of rhodopsin or the corresponding salt bridge between Lys296 and Glu113 in the ligand-free apoprotein opsin^17^ are not unique to rhodopsin or to the family of visual pigments. In the α_1b_ adrenergic receptor, a homologous salt bridge between an aspartate on H3 and a lysine on H7 stabilizes the inactive receptor conformation and is required for constricting the basal activity of the receptor to a low level.^18^ It is broken during agonist-induced receptor activation by interaction of the aspartate with the protonated amine of the catecholamine ligand.^19^ In the δ opioid and the angiotensin AT_1_ receptors interhelical interactions between H3 and H7 are maintained between Asp and Tyr residues and two Asn residues, respectively, which also constrain the basal activity of these receptors.^20–22^

Previous studies have shown that E113Q or E113A opsin mutants are still able to bind 11-*cis* retinal and to form visual pigments, but that these visual pigments have a deprotonated Schiff base and hence absorb in the near-UV around 380 nm.^8^ These UV-absorbing mutants are functional and activate the visual G protein transducin in a light-dependent manner.^9,23^ A protonation of the Schiff base and a concomitant shift of the absorption maximum to the visible range was observed only at very low pH and in the presence of suitable solute anions, with a bound solute anion serving as a counterion to the PSB.^10,24^

One of our main concerns in this study was to shed light on the interplay between Glu113 and Glu181 on EC2 during the transitions to Meta I and Meta II. Invertebrate rhodopsins have Glu181 as a dark state counterion and it was suggested that vertebrate rhodopsins acquired the Glu113 counterion at some point during their evolution.^25^ Recently, a counterion switch model was proposed for the activation pathway of vertebrate rhodopsin.^12^ On the basis of Raman data and mutational data,^26^ Glu181 was suggested to be neutral in the dark state and to become charged by proton transfer to Glu113 in the transition to the active state precursor Meta I, thereby switching the function of the counterion from Glu113 to Glu181. This proton transfer hypothesis was not compatible with Fourier transform infrared (FTIR) spectroscopic data,^27^ and instead a gradual shift of the counterion function from Glu113 to Glu181 on the activation pathway was found, with Glu181 remaining charged during the entire activation process and Glu113 becoming neutralized by proton transfer from the PSB only in the final transition to active Meta II.^11^

In the present study, we examined the activation and deactivation pathways of the E113Q mutant in a native-like membrane environment. To counteract the destabilizing effect of this mutation and to allow reconstitution of the purified receptor into lipid membranes, we used an E113Q mutant with an additionally engineered disulfide bridge in the extracellular domain, which stabilized the folded protein without interfering significantly with receptor activation.^28^ The results indicate clearly that Glu181 cannot serve as a single counterion to the PSB in the absence of the primary Glu113 counterion on the activation pathway of the receptor. The Meta I/Meta II conformational equilibrium, which is maintained in the E181Q mutant,^11^ is absent from the E113Q mutant, which features a deprotonated Schiff base in all photoproduct states up to Meta II. A protonation of the SB is observed only in the transition to Meta III on the deactivation pathway of the receptor. In contrast to wildtype Meta III, the Meta III state of the mutant adopts an active state conformation in a pH-independent manner, indicating the necessity of Glu113 for deactivation of the receptor, but not for re-protonation of the SB, which is achieved presumably by Glu181.

## Results

In this study, we used FTIR spectroscopy, which is a method of choice for investigating the structural changes during activation of rhodopsin due to its excellent sensitivity for changes of both the chromophore and the protein.^29^ Conformational changes of the protein are sensed by the amide I vibrations of the protein backbone, absorbing in the range 1600–1700 cm^− 1^, and by several protonated carboxylic acids, which have their intense C = O stretch absorption in the spectral region 1700–1780 cm^− 1^, and are not crowded by other protein vibrational modes. The position of their C = O stretch is very sensitive to hydrogen bonding within the surrounding functional networks, with positions at around 1770 cm^− 1^, 1740 cm^− 1^, and 1710 cm^− 1^ being indicative for a carboxylic acid making zero, one, or two hydrogen bonds, respectively, with its environment.^30^

With Asp83 and Glu122, rhodopsin has two carboxylic acids in its transmembrane core that take part in two important interhelical networks of the receptor between H1, H2, and H7, and between H3 and H5, respectively^1^ (Fig. 1). Both groups remain protonated throughout receptor activation and can serve as valuable intrinsic reporter groups for conformational changes in their corresponding receptor microdomains. Using these two reporter groups in addition to information derived from other vibrational modes, we can track the path of retinal in its binding pocket during activation of a E113Q mutant and compare it to that in the wildtype receptor.

### The stabilizing N2C/D282C mutation does not significantly affect the activation pathway of rhodopsin

**Fig. 2:**
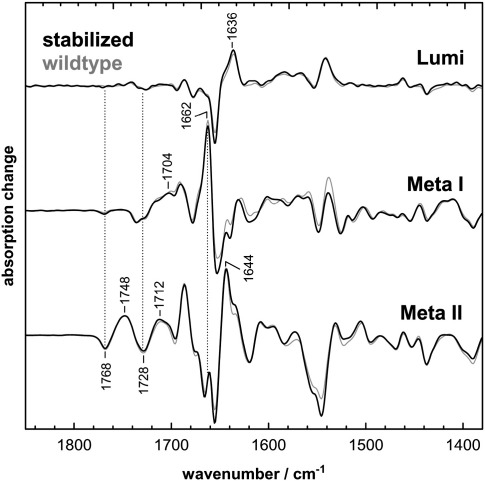
The stabilizing disulfide bridge in the N2C/D282C mutant does not significantly alter the transition from the dark to the photoproduct states. The light-induced transitions of lipid-membrane reconstituted wildtype rhodopsin (gray spectra) and N2C/D282C mutant (black spectra) from the dark state to Lumi (stabilized at - 90 °C, pH 7.0), Meta I (at − 20 °C, pH 7.0), and Meta II (at 10 °C, pH 5.0) reveal essentially identical FTIR difference spectra. This holds in particular in the structurally informative range of protonated carboxylic acids between 1700 cm^−^ ^1^ and 1770 cm^−^ ^1^, and in the amide I range of backbone vibrations around 1650 cm^−^ ^1^.

As a detergent environment profoundly perturbs the conformational equilibrium between Meta I and Meta II, reconstitution of the purified receptor into native-like lipid membranes is essential. As we anticipated an E113Q mutant to be considerably less stable than wildtype rhodopsin, we sought strategies to increase its stability to ensure successful reconstitution. A higher level of stability could prove to be beneficial for crystallographic studies of rhodopsin's inherently unstable active conformation. Such a strategy was devised by the introduction of two cysteines on the N terminus and the extracellular loop 3 in the N2C/D282C mutant, which have been shown to form a disulfide bridge spontaneously (Fig. 1).^28^ This engineered disulfide bridge considerably enhanced the thermal stability of opsin without interfering significantly with pigment formation or pigment activation. Additionally, a recent crystallographic analysis of this stabilized rhodopsin mutant revealed only minor impact of the engineered disulfide bridge on the global fold of the protein.^3^ To further exclude implications of the additional disulfide bridge on the various conformational states of the receptor, we reconstituted the N2C/D282C mutant into phosphatidyl choline (PC) lipid membranes and followed the activation pathway in the transitions from the dark state to the Lumi, Meta I, and Meta II photoproduct states using FTIR difference spectroscopy (Fig. 2). The resulting difference spectra are very similar to those obtained from wildtype rhodopsin in parallel. In particular, the band patterns in the conformationally sensitive range of the amide I vibrations around 1650 cm^− 1^ and of the C = O stretch of protonated carboxylic acids between 1700 cm^− 1^ and 1770 cm^− 1^ are essentially identical. In the transition to the Meta II state, the band pattern due to the hydrogen bonding changes of Asp83 and Glu122 can be observed, leading to a shift of their absorption from 1768 cm^− 1^ and 1728 cm^− 1^, respectively, in the dark state (negative) to a combined Meta II band (positive) at 1748 cm^− 1^. Likewise, proton transfer from the PSB to Glu113 leads to the 1712 cm^− 1^ band of protonated Glu113 in Meta II both in the mutant and wildtype. Therefore, the additional disulfide bond between residues 2 and 282 in N2C/D282C, which is responsible for the reported stabilization, does not interfere significantly with the activation pathway of the receptor.

### The E113Q_stab_ mutant forms a pH-dependent equilibrium between SB and PSB forms

In preliminary experiments, activation of the triple mutant N2C/D282C/E113Q (from hereon E113Q_stab_) was tested in the presence of the detergent dodecyl maltoside (DDM). Meta II minus dark state FTIR difference spectra of E113Q_stab_ correspond to previously published spectra of the (unstabilized) E113A mutant in detergent,^13^ confirming band assignments and results derived in that study also for E113Q_stab_.

**Fig. 3:**
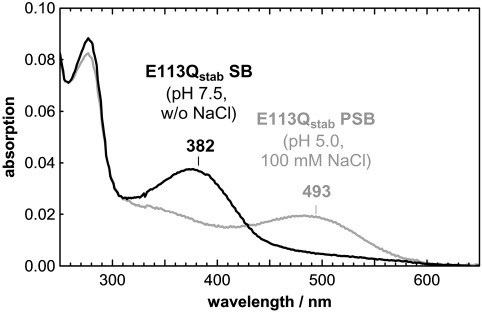
pH- and anion-dependent protonation state of the Schiff base in E113Q_stab_. UV-visible spectra of the E113Q_stab_ mutant reconstituted into PC lipid membranes show an absorption maximum at 382 nm at pH 7.5 in the absence of NaCl, indicating a deprotonated retinal Schiff base. At pH 5.0 in the presence of additional 100 mM NaCl, the absorption maximum is shifted to ∼ 493 nm, indicating a protonated Schiff base.

In order to investigate the properties of E113Q_stab_ in a more native-like membrane environment, the pigment was reconstituted from DDM into PC bilayers. Similar to the (unstabilized) E113Q pigment in detergent,^8,9^ E113Q_stab_ in membranes forms in the dark an equilibrium between a UV-absorbing form with a deprotonated Schiff base, E113Q_stab_ SB (λ_max_ at ∼ 382 nm), and a species with protonated Schiff base, E113Q_stab_ PSB (λ_max_ at ∼ 493 nm). In the absence of chloride, only the SB species is observed at neutral to alkaline pH. At pH 5.0, some contribution of the PSB species is observed, which can be increased considerably by the addition of small amounts of chloride. At pH 5.0 in the presence of 100 mM NaCl, this protonation equilibrium is fully shifted to the side of the PSB form (Fig. 3). This chloride dependence is in full agreement with previous studies of the purified (unstabilized) E113Q mutant,^10,24^ indicating that the stabilizing disulfide bridge in E113Q_stab_ does not interfere with these functional implications of the E113Q mutation.

On the basis of these initial experiments, we prepared E113Q_stab_ SB at pH 7.5 in the absence of NaCl and E113Q_stab_ PSB at pH 5.0 in the presence of 100 mM NaCl.

### The Meta II state of E113Q_stab_

**Fig. 4:**
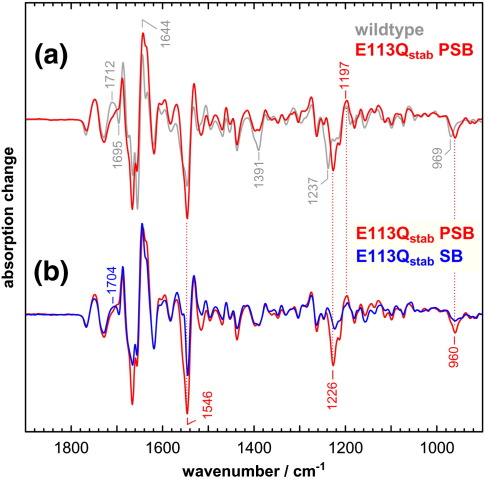
Meta II difference spectra of the E113Q_stab_ mutant. Light-induced Meta II minus dark state FTIR difference spectra are shown for wildtype rhodopsin (gray spectrum in a) and for the E113Q_stab_ mutant in its form with protonated Schiff base (E113Q_stab_ PSB, red spectra in a and b) and deprotonated Schiff base (E113Q_stab_ SB, blue spectrum in b) in lipid membranes. Both protonation states of the mutant pigment form Meta II states with overall features similar to wildtype Meta II, despite the fact that chromophore related bands are partly shifted in the dark state of E113Q_stab_ PSB. Several of these bands have only low intensity or are entirely lacking in the dark state of E113Q_stab_ SB due to the reduced charge alternation of the retinal polyene. Meta II spectra were obtained at 10 °C.

E113Q_stab_ PSB and E113Q_stab_ SB were activated at 10 °C by brief illuminations with orange light (> 530 nm) or 395 nm light from a UV-emitting LED, respectively. Under both conditions, an active Meta II state was formed that revealed the conformational signatures of native Meta II in the FTIR difference spectra (Fig. 4). Additional experiments further confirmed the capability of the activated mutant to interact with G protein-derived peptides (data not shown).

In the Meta II minus dark state difference spectrum of E113Q_stab_ PSB (Fig. 4a), some of the dark state chromophore bands are somewhat downshifted as compared with wildtype, as for example the fingerprint mode at 1226 cm^− 1^ or the hydrogen-out-of-plane (HOOP) mode at 960 cm^− 1^. The intensity of the positive fingerprint mode in Meta II at 1197 cm^− 1^ indicates that there is a partial protonation of the Schiff base in the Meta II state of E113Q_stab_ under these conditions, presumably due to persistent binding of chloride in the retinal-binding pocket of the mutant in Meta II, a behavior found also, to a lesser extent, in native Meta II.^31^ In native pigment, the protonation of Glu113 during the transition to Meta II causes a negative band at 1391 cm^− 1^ for the symmetric COO^−^ stretch of the glutamate in the dark state and a positive band at 1712 cm^− 1^ for the C = O stretch of the glutamic acid in Meta II,^13^ which are absent from the mutant. In E113Q_stab_ there is, however, positive (Meta II) absorption at around 1700 cm^− 1^, which also compensates the negative band in wildtype at 1695 cm^− 1^. A contribution of a carboxylic acid to this band can be excluded from its lack of hydrogen/deuterium (H/D) sensitivity (data not shown). The conformationally sensitive band pattern of Asp83 and Glu122 in the range between 1720 cm^− 1^ and 1770 cm^− 1^ is similar to the wildtype.

The Meta II state of E113Q_stab_ SB has a deprotonated Schiff base. The Meta II minus dark state FTIR difference spectrum differs from that of E113Q_stab_ PSB due mainly to the considerably reduced intensity of chromophore bands, which have little IR intensity when the retinal Schiff base is deprotonated due to the resulting reduced charge alternation along the polyene (Fig. 4b). This is particularly noticeable for the dark state HOOP mode at 960 cm^− 1^, the 1226 cm^− 1^ fingerprint mode, and the 1546 cm^− 1^ ethylenic mode of the chromophore. Further alterations in the range around 1650 cm^− 1^ for both E113Q_stab_ SB and PSB might be accounted for by different positions of the Schiff base C = N stretch mode in the different states.^32^

### The absence of the counterion function of Glu113 in E113Q_stab_ abolishes the pH-dependence of Meta I/Meta II

The pH-dependence of Meta I/Meta II,^15^ which reflects the proton uptake of rhodopsin during its transition to Meta II and is one of the hallmarks of activation of rhodopsin, is absent from E113Q_stab_. Starting from the deprotonated form of E113Q_stab_, we investigated a putative pH-dependence in formation of Meta II both at 10 °C and − 10 °C, which was clearly absent from the accessible pH range up to pH 9, revealing Meta II states at all pH values similar to those shown in Fig. 4b. In contrast to wildtype rhodopsin or the E181Q mutant,^11^ E113Q_stab_ does therefore not have a Meta I state that can be stabilized solely by alkaline pH.

### Lack of the Glu113 counterion alters the Meta II precursor state of E113Q_stab_

**Fig. 5:**
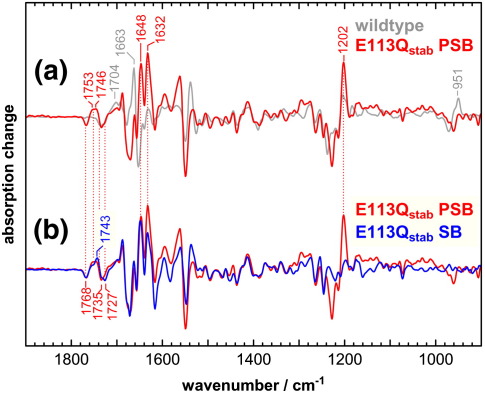
Difference spectra of a cryo-trapped Meta II precursor state of the E113Q_stab_ mutant. Light-induced photoproduct minus dark state FTIR difference spectra of the E113Q_stab_ mutant were recorded at − 30 °C for E113Q_stab_ PSB (red spectra in a and b) and E113Q_stab_ SB (blue spectrum in b) in lipid membranes and are compared to wildtype Meta I minus dark state difference spectra recorded at the same temperature. Similarly to the Meta II difference spectra shown in Fig. 4, deprotonation of the Schiff base in the SB form of E113Q_stab_ reduces the intensity of chromophore-related bands. The Meta II precursor states of both the SB and the PSB forms of the mutant reveal a band pattern of Asp83 and Glu122 between 1720 cm^−^ ^1^ and 1770 cm^−^ ^1^ that is entirely different from that observed in wildtype Meta I and much more similar to Meta II. Also, the Meta I HOOP mode at 951 cm^−^ ^1^ is lacking in the − 30 °C photoproduct of E113Q_stab_ PSB.

**Fig. 6:**
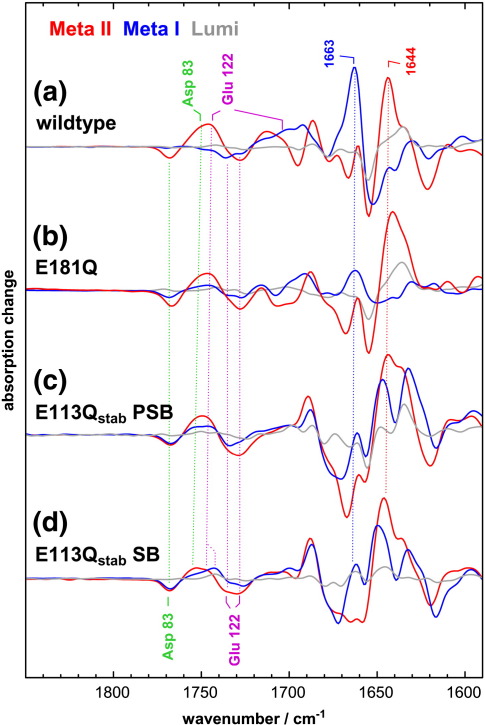
Evolution of structural changes in the Asp83 and Glu122 networks during activation of wildtype rhodopsin and counterion mutants. FTIR difference spectra of the transitions from the dark state to Lumi (gray), Meta I (blue), and Meta II (red) are compared for wildtype rhodopsin (a), the E181Q mutant (b, reproduced from ref. 10), and the PSB (c) and SB (d) forms of E113Q_stab_. These spectra allow us to monitor the changes in the hydrogen bonded networks around Asp83 (green) and Glu122 (purple) and of amide I marker bands of Meta I and Meta II at 1663 cm^−^ ^1^ and 1644 cm^−^ ^1^, respectively. The downshift of Asp83, which is observed in wildtype only in the transition to Meta II, is observed partly in Meta I of E181Q and fully in the photoproduct states at − 30 °C of E113Q_stab_ PSB and SB, as evident from the intensity of the negative peak of Asp83 in the respective dark states. Glu122, which in wildtype is transiently downshifted in Meta I and upshifted in Meta II, experiences in the E113Q_stab_ mutants a Meta II-like upshift already partly in the Meta II precursor state at − 30 °C.

In order to examine the structural properties of Meta II precursor states of the counterion mutant, the photoreaction of E113Q_stab_ was investigated at lower temperature, allowing the cryo-trapping of a Meta II precursor at − 30 °C (Fig. 5). Under these conditions, wildtype rhodopsin produces the inactive Meta I state. Due to the considerable structural differences between this cryo-trapped intermediate of the mutant and native Meta I, we refer to this state as the Meta II precursor state. Similar to Meta I in wildtype, it links the Lumi state of the mutant with its Meta II state (see Fig. 6c and d).

This cryo-trapped Meta II precursor state of E113Q_stab_ PSB (Fig. 5a) has a fully protonated Schiff base, as evident from the strong intensity of the 1202 cm^− 1^ fingerprint mode of the chromophore. The lack of the Meta I HOOP mode at 951 cm^− 1^ indicates a chromophore with an essentially planar polyene in contrast to wildtype Meta I. In the amide I range, the cryo-trapped Meta II precursor state of E113Q_stab_ PSB shows pronounced positive bands at 1632 cm^− 1^ and 1648 cm^− 1^, which merge at higher temperature into the Meta II marker band at 1644 cm^− 1^. The Meta I amide marker band of wildtype at 1663 cm^− 1^, on the other hand, is completely lacking in the mutant. In the range of the protonated carboxylic acids, we observe in wildtype a downshift of the band of Glu122 from a doublet at 1735 cm^− 1^ and 1728 cm^− 1^ in the dark state to 1704 cm^− 1^ (in a broader shoulder), indicating a strengthening of its hydrogen bonding in its interhelical network with His211 and Trp126 (see Figs. 1 and 6). Conversely, in the cryo-trapped Meta II precursor state of E113Q_stab_ PSB, Glu122 is upshifted by about half of of its upshift in Meta II, indicating a partial disruption of its hydrogen bonding in this precursor state. Asp 83, which undergoes only slight changes in the transition to Meta I in wildtype, experiences in E113Q_stab_ a downshift from 1768 cm^− 1^ to 1753 cm^− 1^ comparable to that in Meta II, indicating a full rearrangement of the involved H1/H2/H7 network to an active state conformation in this Meta II precursor state at − 30 °C.

The cryo-trapped Meta II precursor state of E113Q_stab_ SB (Fig. 5b) maintains its deprotonated Schiff base and therefore again lacks intensity in the chromophore bands due to the lack of charge alternation along the SB chromophore. The presence of an SB in this state was also verified using UV-visible spectroscopy (data not shown). Conformationally sensitive bands of amide I vibrations and carboxylic acids are very similar to the Meta II precursor state of the PSB form of E113Q_stab_, despite a slightly smaller upshift of the position of Glu122.

### The Meta III states on the deactivation pathways of wildtype rhodopsin and E113Q_stab_ SB

**Fig. 7:**
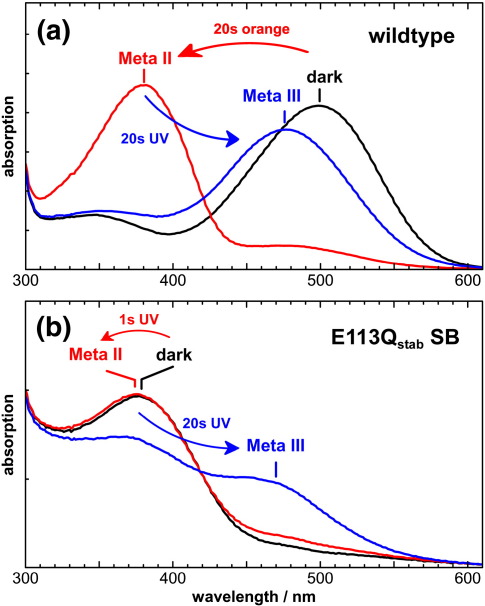
The transition to the Meta III state of E113Q_stab_ SB involves a protonation of the Schiff base. (a) The dark state of wildtype rhodopsin (λ_max_ 500 nm) is converted by orange illumination (> 530 nm) to Meta II (380 nm), which can be converted to Meta III (470 nm) by UV illumination (∼ 395 nm). (b) The dark state of E113Q_stab_ SB (382 nm) is converted by a 1 s UV light pulse to Meta II, which is slightly blue-shifted. Extended UV-illumination (20 s) converts Meta II to Meta III, involving protonation of the Schiff base and an absorption shift to ∼ 470 nm, similar to the wildtype. This protonation of the Schiff base of E113Q_stab_ SB indicates a counterion different from Glu113 in the Meta III state. As evident from the slight absorption increase at 470 nm in the red spectrum, a small amount of Meta III is formed during the first 1 s UV light pulse. Experiments were performed at 10 °C and pH 5.0 in the case of wildtype and at pH 7.5 in the case of E113Q_stab_ SB, tickmarks correspond to 50 mOD.

In wildtype rhodopsin, the active state conformation of Meta II decays in the transition to Meta III, which is triggered by a thermal isomerization of the Schiff base C15 = N double bond from all-*trans* 15-*anti* to all-*trans* 15-*syn*.^33^ This deactivation step can likewise be triggered by UV-illumination of the Meta II state.^34^ The transition to Meta III involves a re-protonation of the SB and a shift of the absorption maximum to 470 nm (Fig. 7a). As shown earlier, the transition to Meta III regularly involves a deactivation of the receptor at neutral to alkaline pH, while at pH 4.0, Meta III adopts an active conformation similar to that of Meta II.^35^

In E113Q_stab_ SB, photolysis of the UV-absorbing pigment with a short (1 s) UV light pulse converts the dark state of the mutant to its Meta II state, which is slightly blue-shifted as compared to the dark state (Fig. 7b), in agreement with time-resolved UV-visible studies.^36^ Further UV illumination of this Meta II state for 20 s converts Meta II to a photoproduct with PSB absorbing at 470 nm, similar as observed above for wildtype rhodopsin.

The quantum yield of the secondary reaction from Meta II to Meta III is astonishingly high. The 1 s illumination photo-converts about 40–50% of the dark state (see below), of which ∼ 80% are present as Meta II, while 20% were further photo-converted to Meta III, as evident from the small absorption increase at 470 nm in the red spectrum of Fig. 7b. The quantum yield of this secondary reaction is therefore roughly half of that of the primary reaction. In order to avoid complications with the partially protonated Schiff base of Meta II of E113Q_stab_ PSB, we focus on the Meta III state of E113Q_stab_ SB stabilized in the absence of solute chloride.

### FTIR characterization of Meta III of E113Q_stab_ SB

**Fig. 8:**
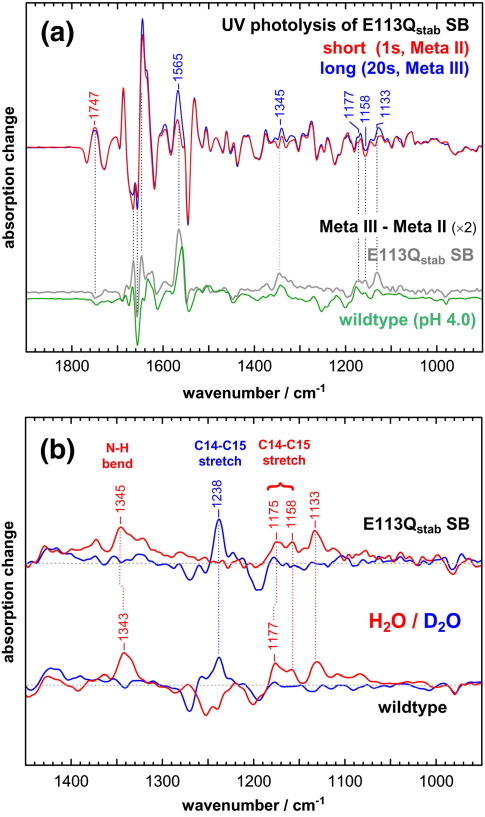
The Meta III state of E113Q_stab_ adopts an active state conformation. (a) The conversion of the dark state of E113Q_stab_ SB to its Meta II and Meta III states was achieved as in Fig. 7 at 10 °C and pH 7.5 by a sequence of short and long periods of UV illumination and followed in FTIR difference spectra Meta II minus dark state in red and Meta III minus dark state in blue. Spectra were normalized to account for differing pigment conversion. As evident from the similarity of the spectra in the conformationally sensitive range between 1600 cm^−^ ^1^ and 1800 cm^−^ ^1^, the Meta III state of E113Q_stab_ retains the active state conformation of Meta II. The strong band at 1565 cm^−^ ^1^ is the ethylenic C = C stretch of the Meta III chromophore, reflecting the protonation of the Schiff base. The Meta III minus Meta II difference spectrum of E113Q_stab_ (gray) corresponds in many aspects to that of wildtype (green) obtained at pH 4.0, where the active conformation of Meta III is stabilized, with some differences in the range between 1600 cm^−^ ^1^ and 1700 cm^−^ ^1^. (b) The signature of the all-*trans* 15-*syn* chromophore of Meta III in E113Q_stab_ is identified in Meta III minus Meta II difference spectra of E113Q_stab_ obtained in H_2_O (red) and ^2^H_2_O (blue). This signature consists of an NH bending mode at 1345 cm^−^ ^1^ coupled to the C14–C15 stretch modes at 1175 cm^−^ ^1^ and 1158 cm^−^ ^1^ in H_2_O, and a > 50 cm^−^ ^1^ upshift of the latter to 1238 cm^−^ ^1^ in ^2^H_2_O, where this coupling is absent. A very similar signature is observed for Meta III of wildtype, which is shown here again for the active Meta III conformation adopted at pH 4.0.^35^

In Fig. 8a, the light-induced Meta III formation of E113Q_stab_ SB is monitored in the Meta III minus dark state FTIR difference spectrum (blue) under conditions identical with those in Fig. 7b, which is compared with the Meta II minus dark state (red) difference spectrum obtained by brief illumination. While the 20 s illumination used to produce Meta III is sufficient to establish a photo-stationary state, the first 1 s UV light pulse to produce mainly Meta II leads to pigment conversion of only 40∼50%. The spectra in Fig. 8a were therefore normalized for easier comparison using band patterns of the dark state. As evident from the almost identical band pattern in the conformationally sensitive range between 1600 cm^− 1^ and 1800 cm^− 1^, Meta III of the mutant adopts an active state protein conformation very similar to that of Meta II. Note that the photoproduct state in the Meta III minus dark state spectrum presumably still contains residual Meta II, as indicated by the small absorption peak at 380 nm remaining in the blue spectrum of the photostationary state in Fig. 7b. However, this does not alter the above conclusion.

By subtracting the Meta II minus dark state spectrum from the Meta III minus dark state spectrum, we obtain the Meta III minus Meta II difference spectrum of the mutant (Fig. 8a, gray spectrum). This spectrum is very similar to the corresponding double difference spectrum of wildtype (green spectrum) obtained under conditions where wildtype Meta III adopts a similarly active conformation (pH 4.0).^35^ The mutant spectrum reveals a pronounced Meta III band at 1565 cm^− 1^ of the ethylenic C = C stretch mode of the chromophore, reflecting the protonation of the SB in Meta III, and a series of other chromophore bands between 1100 cm^− 1^ and 1400 cm^− 1^ being indicative of the isomerization of the Schiff base to 15-*syn*, which is addressed below. As seen in the wildtype spectrum, there is a slight decrease of the absorption of the protonated carboxylic acid Glu122 (H3) at 1747 cm^− 1^, which is positioned next to the ring of retinal. This residue is therefore slightly altered by the C15 = N isomerization of the chromophore, but retains its overall Meta II-like absorption pattern. Slight changes in the band pattern between 1600 cm^− 1^ and 1700 cm^− 1^ in the mutant as compared to wildtype are due, in part, to contributions of the amide absorption of the glutamine at position 113 in the mutant.

The all-*trans* 15-*syn* isomer can be identified by the strong coupling between the C14–C15 stretching modes and the Schiff base NH bending mode in a 15-*syn* PSB,^37^ which is abolished in ^2^H_2_O, leading to a very pronounced upshift of the C14–C15 modes by more than 50 cm^− 1^.^33^ Comparison of the Meta III minus Meta II difference spectrum of E113Q_stab_ SB with that of wildtype (again for the active conformation of wildtype Meta III stabilized at pH 4.0) reveals almost identical positions of the NH bending mode at 1345 cm^− 1^ (Fig. 8b). The C14–C15 stretch absorbs at 1175 cm^− 1^ and 1158 cm^− 1^ in H_2_O, and experiences a pronounced upshift to 1238 cm^− 1^ in ^2^H_2_O. The vibrational modes containing C14–C15 contributions have been identified in wildtype using ^13^C isotopic labeling of C14–C15,^33,35^ the H/D-sensitive band at 1133 cm^− 1^ being unresponsive to the labeling.

### The receptor conformation in Meta III of E113Q_stab_ SB and wildtype rhodopsin

**Fig. 9:**
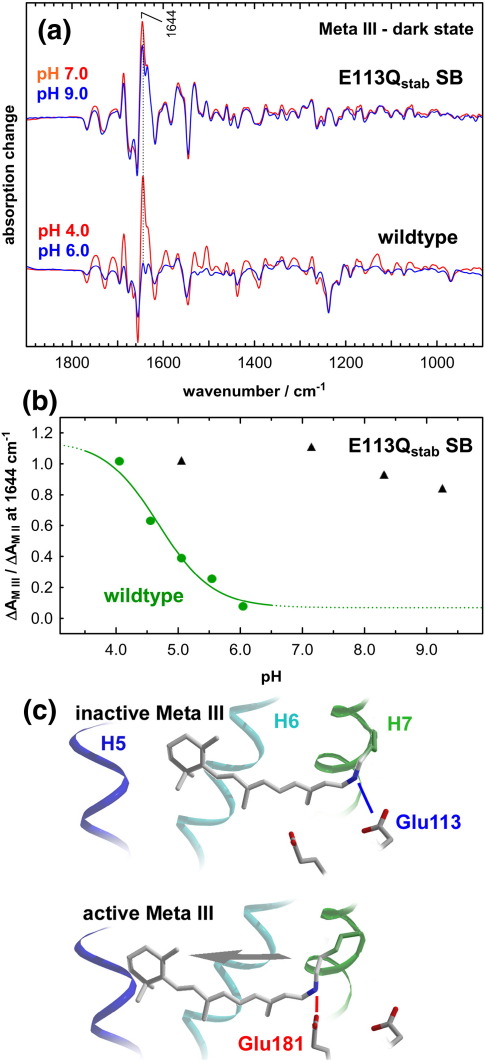
The Glu113/PSB salt bridge is responsible for receptor deactivation in the transition to Meta III. (a) Meta III minus dark state FTIR difference spectra were generated by orange and UV illumination in the case of wildtype rhodopsin or by a single 20 s UV illumination in the case of the UV-absorbing E113Q_stab_ mutant at 10 °C at different pH values. (b) Meta III of wildtype rhodopsin shows a pH-dependent transition from an active Meta II-like conformation at extremely acidic pH to an inactive conformation at neutral pH with an apparent p*K*_A_ of 4.7 as reported.^35^ This pH-dependent transition is followed here by monitoring the intensity of the active state marker band at 1644 cm^−^ ^1^ in Meta III minus dark state difference spectra, normalized by its intensity in Meta II minus dark state reference spectra. Meta III of the E113Q_stab_ mutant, on the other hand, persists in the active conformation also in the very alkaline pH range. Re-formation of the salt bridge between the PSB and Glu113 is therefore responsible for the deactivation of the receptor in the transition to Meta III at neutral to alkaline pH. (c) In this cartoon of the retinal-binding pocket of the Meta III states of wildtype rhodopsin, the inactive conformation of Meta III is stabilized by the structural salt bridge between the PSB and Glu113, which keeps the retinal in a position similar as in the inactive dark state or Meta I. In the active conformation of Meta III the PSB is translocated along the long axis of retinal away from Glu113 toward Glu181, similar as reported previously for Meta II.^38^ This allows for formation of a salt bridge with Glu181, which stabilizes the PSB but does not constrain the receptor in an inactive conformation (the cytoplasmic side points upward).

Wildtype Meta III exists in a pH-dependent conformational equilibrium between an active conformation at acidic pH and an inactive conformation at pH 6.0 and above,^35^ which is evident from the FTIR difference spectra Meta III minus dark state produced by consecutive orange and UV illuminations (Fig. 9a). The pH-dependence of this equilibrium can, for instance, be monitored using the intensity of the amide I marker band of the active state conformation at 1644 cm^− 1^, revealing a titration curve following a regular Henderson–Hasselbalch curve with an apparent p*K*_A_ of 4.7 (at 10 °C) (Fig. 9b). Meta III of the E113Q_stab_ mutant, on the other hand, adopts an active state conformation over the entire pH range up to very alkaline values. This pH-independence of the active state conformation of Meta III in the mutant indicates that Glu113 is responsible for stabilizing the inactive conformation of Meta III in wildtype by re-forming a salt bridge to the PSB.

## Discussion

Besides the shape of the 11-*cis* retinal ligand, the salt bridge between the positively charged PSB and its negative protein counterion, Glu113 on H3, is the second major conformational constraint that stabilizes the inactive dark state of rhodopsin and that needs to be broken during activation of the receptor. A similar salt bridge exists in opsin between Glu113 and Lys296 on H7 (which forms the covalent Schiff base with retinal in the pigment),^17^ and between homologous residues in the α-adrenergic receptor,^18^ and is important for restraining basal activity of these receptors. In rhodopsin, breaking of this salt bridge in the transition to the signaling state, Meta II, is connected with a movement of retinal along its long axis toward H5,^38^ disrupting a hydrogen bonded interhelical network of His211 with Glu122 and Trp126 on H3,^39,40^ and triggering via interaction with Trp265^41^ an outward-directed rigid body movement of H6 (Fig. 1).^42,43^ This concerted motion of retinal and transmembrane helices is also coupled to conformational changes in a hydrogen bonded interhelical network between H1, H2, and H7, formed by Asn55 (H1), Asp83 (H2), Asn302 (H7), and other residues, as well as structural water molecules.

The neutral carboxylic acids Asp83 and Glu122 participate in the H1/H2/H7 and H3/H5 microdomains, respectively, and serve as valuable site-specific reporter groups for infrared spectroscopy. The stretching vibration of their C = O function absorbs in a relatively uncrowded spectral region between 1700 cm^− 1^ and 1780 cm^− 1^ and is highly sensitive to changes in their hydrogen bonding resulting from conformational transitions in the surrounding networks. Using these two reporter groups, we can follow the evolution of conformational changes within the associated two microdomains during receptor activation and correlate them with molecular changes detected in other domains. In Fig. 6a, the photoproduct minus dark state FTIR difference spectra of the transition to the inactive Lumi state trapped at − 90 °C, to Meta I trapped at − 30 and at − 20 °C, and to active Meta II stabilized at 10 °C are shown for the wildtype receptor.

Asp83 (marked green in Fig. 6) reports only very slight changes in the H1/H2/H7 network in the transitions from the dark state to Lumi and Meta I, and a major change in the transition to Meta II, downshifting the absorption of Asp83 by approximately 20 cm^− 1^, corresponding to the net formation of one hydrogen bond in the rearrangement of the microdomain in the active receptor.^30^ Glu122 (marked purple in Fig. 6) in the H3/H5 microdomain is in contact with the ring of retinal and has two distinct negative absorption peaks in the dark, probably reflecting two different populations of ring isomers in the dark state.^44^ In this state, Glu122 makes two hydrogen bonds of intermediate strength, one to the backbone carbonyl of His211 on H5 and an intrahelical hydrogen bond to the ring of Trp126 (Fig. 1).^1,2,4^ While this hydrogen bonding is not altered substantially in the transition to Lumi, it is transiently strengthened in Meta I, leading to a downshift of the Glu122 absorption to 1704 cm^− 1^.^40^ In the transition to active Meta II, the absorption of Glu122 is considerably upshifted to above 1740 cm^− 1^,^45^ presumably reflecting the disruption of the hydrogen bond to the backbone carbonyl of His211, that is also detected by NMR.^39^

Turning to the E113Q_stab_ mutant in either Schiff base protonation form (Fig. 6c and d), neither Asp83 nor Glu122 show pronounced changes in Lumi, indicating that at − 90 °C re-arrangements of helices are largely inhibited. At − 30 °C, however, in the transition to a Meta II precursor state, Asp83 experiences in E113Q_stab_ a downshift of its absorption that is observed in wildtype in a similar fashion only in the transition to Meta II. The absence of the salt bridge between H3 and the PSB, which is linked to H7 by the side chain of Lys296, allows the H1/H2/H7 network to adopt its active state conformation already in this precursor state stabilized at − 30 °C by uncoupling it from other microdomains of the receptor. Also, Glu122 experiences in this precursor state conformational changes very different from native Meta I. Instead of a strengthening of its hydrogen bonding pattern as in wildtype Meta I, we observe a weakening by about half of that observed later in the transition to Meta II. As Glu122 is in contact with the ring of retinal, this indicates an interaction of the ring with the H3/H5 network that is considerably altered in this precursor state of the mutant as compared to wildtype, and partly (∼ 50%) Meta II-like. This indicates that a partial movement of the ligand toward H5 occurs at − 30 °C, and is responsible for this alteration of the activation pattern in the mutant.

These changes in the activation pathway of the E113Q_stab_ mutant sensed by the Asp83 and Glu122 reporter groups are reflected in the amide I pattern of the protein backbone. The amide I marker band of Meta I at 1663 cm^− 1^ (Fig. 6a) is developed only poorly in the spectra of E113Q_stab_ at − 30 °C (Fig. 6c and d, blue) and instead two intense amide I bands are observed at lower wavenumbers that merge at 10 °C into the Meta II marker band complex centered at 1644 cm^− 1^.

It is instructive to compare this impact of mutational neutralization of Glu113 with that of Glu181 on EC2. In native rhodopsin, Glu181 was proposed to contribute in the dark state to a complex counterion with Glu113,^11^ and was reported to assume a major part of the counterion function in Meta I.^12^ In Fig. 6b, we compare the conformational evolution of the active state of E181Q with that of E113Q_stab_ and wildtype rhodopsin (see Ref. 11 for a more thorough analysis). Also in E181Q, there is a partial anticipation of a Meta II-like conformation of the H1/H2/H7 network as sensed by Asp83 at − 20 °C, although not as complete as in E113Q_stab_. The amide I marker band of Meta I at 1663 cm^− 1^ is decreased similarly in Meta I of E181Q and E113Q_stab_, while a concomitant increase in the range of the Meta II marker band around 1644 cm^− 1^ is lacking in Meta I of E181Q, in contrast to the Meta II precursor state of E113Q_stab_ stabilized at − 30 °C.

The structural importance of the salt bridge of the PSB to Glu113 becomes most obvious on the background of the activation pattern in E113Q_stab_ PSB, where a bound solute anion such as chloride compensates for the loss of the negative charge in the stabilization of a protonated Schiff base. This compensation by the bound anion is merely electrostatic in nature, increasing the apparent p*K*_A_ of the Schiff base by the presence of a negative countercharge. This bound anion has no discernible structural relevance and cannot mimic the structural role of the negatively charged sidechain of Glu113. This becomes evident in the almost identical spectral evolution during activation of E113Q_stab_ SB and E113Q_stab_ PSB (Fig. 6c and d). Even the small residual differences between both forms of E113Q_stab_ are possibly a consequence of a different polarization of the retinal-binding pocket in the two forms rather than of a different structure.

Can Glu181 on EC2 compensate for Glu113? Certainly not in the dark state, as evident from the lack of protonation of the Schiff base in the absence of suitable solute anions.^8,9^ On the basis of the results obtained with the E181Q mutant, the function of the counterion was suggested to shift, at least partially, to Glu181 in the transition to Meta I.^11,12^ While the E181Q mutant maintained the pH-dependent conformational equilibrium between the Meta I and Meta II states,^11^ this equilibrium is shown here to be absent from the E113Q mutant. This is presumably due to a considerable lowering of the enthalpy change of the transition from Meta I to Meta II by the mutational neutralization of the PSB salt bridge, such that this step becomes independent of the cytoplasmic proton uptake reaction. Even in the Meta II precursor state of E113Q_stab_, which can be cryo-trapped at − 30 °C, the conformation of the receptor is considerably more evolved toward an active state conformation than in the Meta I state of E181Q stabilized at a similar temperature, as evident from the conformational changes in the H1/H2/H7 microdomain and the amide I marker band analysis. Notably, this distinction holds for both E113Q_stab_ SB and PSB.

As shown in this and in previous time-resolved UV-visible studies,^36^ the SB forms of E113Q mutants of rod rhodopsin do not produce transient photo-intermediates with a PSB on the main activation pathway. This implicates a quite different electrostatic environment along the path of the Schiff base in the E113Q mutant of rod rhodopsin as compared with the mouse UV cone pigment or the E113Q mutant thereof, where a protonation of the SB in Lumi and Meta I, respectively, has been reported.^46,47^

In the photoproducts of the UV-absorbing SB form of E113Q_stab_, a protonation of the SB is observed only in the transition from Meta II to Meta III, triggered by C15 = N Schiff base isomerization of the chromophore from all-*trans* 15-*anti* to all-*trans* 15-*syn*.^33^ This indicates that a counterion different from Glu113 stabilizes the PSB in this state. As these experiments were performed in the absence of small solute anions such as chloride, and using bis-tris-propane and Mes buffers with relatively bulky anions, a Meta III-specific binding of a solute anion near the Schiff base appears unlikely. Glu181 as the only ionizable residue in the vicinity of the PSB is therefore the most likely candidate for this counterion function in the absence of Glu113. Does this interaction, however, have similar structural implications as the salt bridge between Glu113 and the PSB, which stabilizes inactive receptor conformations? In wildtype rhodopsin, Meta III exists in a conformational equilibrium between active and inactive conformations, which is on the side of the inactive conformation except for the very acidic pH range. Both conformations have a PSB, despite the fact that Glu113 becomes protonated in the active, low-pH Meta III state, as evident from its FTIR protonation signature.^35^ In E113Q_stab_, only the active conformation of Meta III is observed, which indicates that Glu113 is the structural counterion that stabilizes the inactive conformation of Meta III in wildtype.

While Glu181 appears to be capable of stabilizing a PSB in the active conformation of Meta III in wildtype and similarly in the E113Q mutant, this interaction obviously does not exert the same constraints as the Glu113/PSB salt bridge and is not able of keeping the receptor in an inactive conformation. The PSB in the all-*trans* 15-*syn* Meta III isomers can therefore be stabilized either by Glu113 or Glu181, but these two interactions have entirely different structural implications. While formation of the Glu113/PSB salt bridge is responsible for a deactivation of the receptor in the inactive conformation of the Meta III conformational equilibrium, the putative interaction between the PSB and Glu181 is clearly compatible with an active state conformation.

These findings are depicted in the structural cartoon shown in Fig. 9c. Glu181 is about 7 Å distant from Glu113 in the dark state crystal structures. The suggested switch of the counterion requires significant movement of the all-*trans* 15-*syn* PSB from Glu113 toward Glu181 in the transition from the inactive to the active conformation of Meta III. Such a translational movement of retinal of approximately 4 Å was shown to take place during receptor activation in the transition to Meta II in solid-state NMR experiments.^38^ In the active conformation of Meta III, the retinal all-*trans* 15-*syn* isomer presumably occupies a position quite similar to that of the all-*trans* 15-*anti* isomer in Meta II. This is supported by the fact that only small changes are observed for the absorption pattern of Glu122, positioned next to the ring of retinal, in the transition from Meta II to the active Meta III state.

The p*K*_A_ of the 15-*anti* Schiff base in Meta II had been reported to be below 2.5 in the absence of small solute anions.^31^ In the active conformation of Meta III, on the other hand, the protonated form of the 15-*syn* Schiff base is stable at least up to pH 9, as probed in the E113Q_stab_ mutant, despite the fact that the conformational transitions involved in the transition from Meta II to active Meta III appear to be quite small. This large shift of the p*K*_A_ of the Schiff base must therefore be achieved mainly by the re-orientation of the Schiff base induced by the C15 = N isomerization rather than by a large conformational transition of the receptor. As a consistent model, we propose in Meta II an orientation of the 15-*anti* Schiff base toward the cytoplasmic side of the receptor, away from Glu181, resulting in a low apparent p*K*_A_, while the 15-*syn* Schiff base in active Meta III is oriented toward Glu181, thereby increasing the apparent p*K*_A_ considerably. The isomerization of the C15 = N Schiff base double bond triggers this p*K*_A_ switch by translocating the Schiff base between different environments. Such a rapid shift of p*K*_A_ induced by C15 = N isomerization is consistent with time-resolved UV-visible and FTIR experiments of the Meta II to Meta III transition in wildtype.^48^ Those experiments indicate a fast re-protonation of the Schiff base by a local change with a time constant of less than 5 ms. This is followed on a much longer timescale of more than 1 s by the slower global conformational changes responsible for the deactivation of the receptor in the transition to the inactive Meta III conformation.

UV light-induced isomerization of the C15 = N Schiff base double bond in Meta II appears to depend on the deprotonated form of the all-*trans* retinal Schiff base and is not observed in a Meta II_PSB_ state, in which a protonation is stabilized by the presence of suitable solute anions.^49^ As rhodopsin has a protonated retinal Schiff base in the dark state, with an absorption maximum at 500 nm, it can be photoactivated by green light, which triggers the 11-*cis* 15-*anti* to all*-trans* 15-*anti* isomerization of the chromophore but does not cause further 15-*anti* to 15-*syn* isomerization in Meta II absorbing at 380 nm. The UV-absorbing E113Q counterion mutant and similarly UV-absorbing cone pigments, which have an SB in the dark state, require UV light for photoactivation. Lengthy UV illumination may induce such secondary 15-*anti* to 15-*syn* isomerization in the respective Meta II states (and possibly also earlier states), as shown here explicitly for the E113Q_stab_ mutant. In this mutant pigment, the C15 = N isomerization is accompanied by protonation of the SB and thus easily detectable. UV-absorbing cone pigments may have a different electrostatic environment in their chromophore-binding pockets, such that C15 = N isomerization of the SB does not necessarily lead to the same upshift of p*K*_A_. Such secondary isomerizations may therefore go undetected by UV-visible spectroscopy.

## Conclusion

The study of the E113Q mutant of rhodopsin offers interesting insight into the interplay between Glu113 on H3 and Glu181 on EC2 in their contribution to the counterion function in the dark and the photoproduct states. Glu181 contributes to the salt bridge between the PSB and Glu113, and thereby to the extraordinarily high level of stability of the dark state. During receptor activation, this salt bridge needs to be broken to allow re-arrangement of the transmembrane helices, which appears to happen in a stepwise manner by a gradual shift of the counterion function from Glu113 to Glu181 in Meta I, leading to a complete disruption in Meta II. Mutational neutralization of the PSB salt bridge in the E113Q_stab_ mutant leads to a considerably altered activation pathway, in which the classical pH-dependent conformational equilibrium between Meta I and Meta II is absent. Using FTIR difference spectroscopy and specific reporter groups in different microdomains, the interhelical hydrogen-bonded network between helices 1, 2, and 7 could be further shown to adopt an active state conformation already in a Meta II precursor state cryo-trapped at − 30 °C. In the E113Q mutant, Glu181 can therefore not compensate for the lack of Glu113 on the activation pathway. A protonation of the SB is observed only in the transition to Meta III involving an isomerization of the Schiff base C15 = N double bond, which is presumably stabilized by Glu181. This interaction is, however, not sufficient for a deactivation of the receptor, underlining the unique role of the PSB salt bridge to Glu113 in constraining the receptor in an inactive conformation.

## Materials and Methods

### Sample preparation

The stabilized rhodopsin N2C/D282C and the E131Q_stab_ mutant N2C/D282C/E113Q were prepared by transient expression in COS or HEK293 cells, combination with 11-*cis* retinal chromophore, and purification on a 1D4 antibody column in 0.02% *n*-dodecyl-β-D-maltoside (DDM) as described.^3,28^ Both mutants were further reconstituted into lipid membranes. Initial attempts to purify E113Q_stab_ in the easily dialyzable detergent β-octyl glucoside was not successful, recalling the importance of the PSB-Glu113 salt bridge for structural stability of the pigment. We therefore reconstituted pigment purified in the more stabilizing detergent DDM into egg PC membranes with a lipid to protein molar ratio of 200:1. Reconstitution was achieved by extended dialysis over six days at 4 °C with a 7 kDa cutoff against 1 mM sodium phosphate buffer, pH 6.5, with frequent buffer changes. The resulting proteoliposomes were collected by centrifugation. Wildtype rhodopsin was prepared from bovine retinae, purified on a concanavalin A column in 0.02% DDM, and reconstituted into PC membranes according to published procedures^50,51^ in parallel with the E113Q_stab_ mutant pigment to ensure identical sample conditions.

Spectra from the E181Q mutant in PC membranes were obtained as described.^11^

FTIR and UV-visible experiments were performed with sandwich samples described in detail elsewhere,^29^ with 0.1–0.2 nmol of recombinant pigment. Samples were equilibrated with 40 μl of buffer before sandwiching them with the top window. E113Q_stab_ SB samples were prepared with 200 mM bis-tris-propane buffer at pH 7.5, while E113Q_stab_ PSB samples were prepared with 100 mM Mes buffer and 100 mM NaCl at pH 5.0. Samples with wildtype pigment were prepared correspondingly with pH 5.0 for Meta II experiments and with pH 7.5 otherwise. Meta II and Meta III spectra were measured at 10 °C, Meta I spectra at − 30 °C (− 20 °C for E181Q and wildtype), Lumi at − 90 °C, and Batho at − 183 °C. For H/D exchange, sample films were equilibrated with ^2^H_2_O and dried under nitrogen before adding the respective buffer prepared in ^2^H_2_O.

### Spectroscopy

FTIR-difference spectroscopy was performed with a Bruker Vertex FTIR spectrometer as described.^52^ Spectra were accumulated over 1 min using a liquid nitrogen-cooled mercury-cadmium-telluride (MCT) detector at a spectral resolution of 4 cm^− 1^. Samples were photolyzed either for 10 s with a 150 W tungsten lamp via a fiber optic and a 530 nm long-pass filter (for wildtype rhodopsin, stabilized rhodopsin, and E113Q_stab_ PSB) or by a 1 s near-UV illumination using a single 10 mW UV LED (Yoldal CO. Ltd., Taiwan) with an emission peak at 395 nm (for E113Q_stab_ SB). For generation of the Meta III state of E113Q_stab_ SB, the UV illumination was extended to 20 s. The temperature of the samples was controlled by a circulating water-bath, a Peltier cooling unit, or a liquid nitrogen-operated cryostat, depending on the desired temperature. UV-visible spectroscopy was performed with identical samples in a Perkin-Elmer Lambda 17 spectrophotometer equipped with a temperature-controlled sample holder. Illumination conditions were identical with those for the FTIR experiments.

### Molecular models

Molecular models are based on the coordinates of the dark state PDB 1GZM,^4^ of the dark state of the stabilized N2C/D282C mutant pdb 2J4Y,^3^ and of the Lumi state 2HPY,^6^ and were prepared with software Deep View 3.7^53^‡ and POV Ray 3.5§.

## Footnotes

‡ http://www.expasy.org/spdbv

§ http://www.povray.org
